# Probiotic triangle of success; strain production, clinical studies and product development

**DOI:** 10.1093/femsle/fnaa167

**Published:** 2020-10-13

**Authors:** Sofia D Forssten, Arja Laitila, Johanna Maukonen, Arthur C Ouwehand

**Affiliations:** DuPont Nutrition & Biosciences, Sokeritehtaantie 20, 02460 Kantvik, Finland; DuPont Nutrition & Biosciences, Sokeritehtaantie 20, 02460 Kantvik, Finland; DuPont Nutrition & Biosciences, Sokeritehtaantie 20, 02460 Kantvik, Finland; DuPont Nutrition & Biosciences, Sokeritehtaantie 20, 02460 Kantvik, Finland

**Keywords:** probiotics, *Lactobacillus*, *Bifidobacterium*, health benefit, strain production, product development

## Abstract

The successful development of probiotic foods and dietary supplements rests on three pillars; each with their specific challenges and opportunities. First, strain production; this depends on selecting the right strain with promising technological properties and safety profile. Further the manufacturing of the strain in a stable format at sufficiently high yield, following regulatory and customer requirements on culture media ingredients and other processing aids. The second pillar are the preclinical and clinical studies to document that the strain is a probiotic and exerts a health benefit on the host, the consumer. Especially when aiming for a regulator approved health claim, clinical studies need to be thoroughly performed; following appropriate ethical, scientific and regulatory guidelines. Finally, the probiotic will need to be incorporated in a product that can be brought to the consumer; a dietary supplement or a functional food. Because of the live nature of probiotics, specific challenges may need to be dealt with. Although experience from other strains is helpful in the process, the development is strain specific. Commercialisation and marketing of probiotics are strictly but differently regulated in most jurisdictions; defining what can and cannot be claimed.

## Teaser

Successful development of probiotics relies on production of a probiotic strain, documenting it's health benefits and inclusion in a consumer product.

## INTRODUCTION

Health and wellness continue to be key consumer trends with a sustained interest in health maintenance and health support solutions. The revolution in intestinal microbiota analyses has also reached the popular media, consumers are therefore becoming increasingly aware of the crucial role the microbiota plays in wellbeing; also, beyond the intestine. In this dynamic environment, probiotics gain importance and have even led to recommendations by learned societies on their use e.g. the World Gastroenterology Organization and the American Gastroenterological Association (Guarner *et al*. 2017; Su *et al*. 2020).

**Figure 1.**
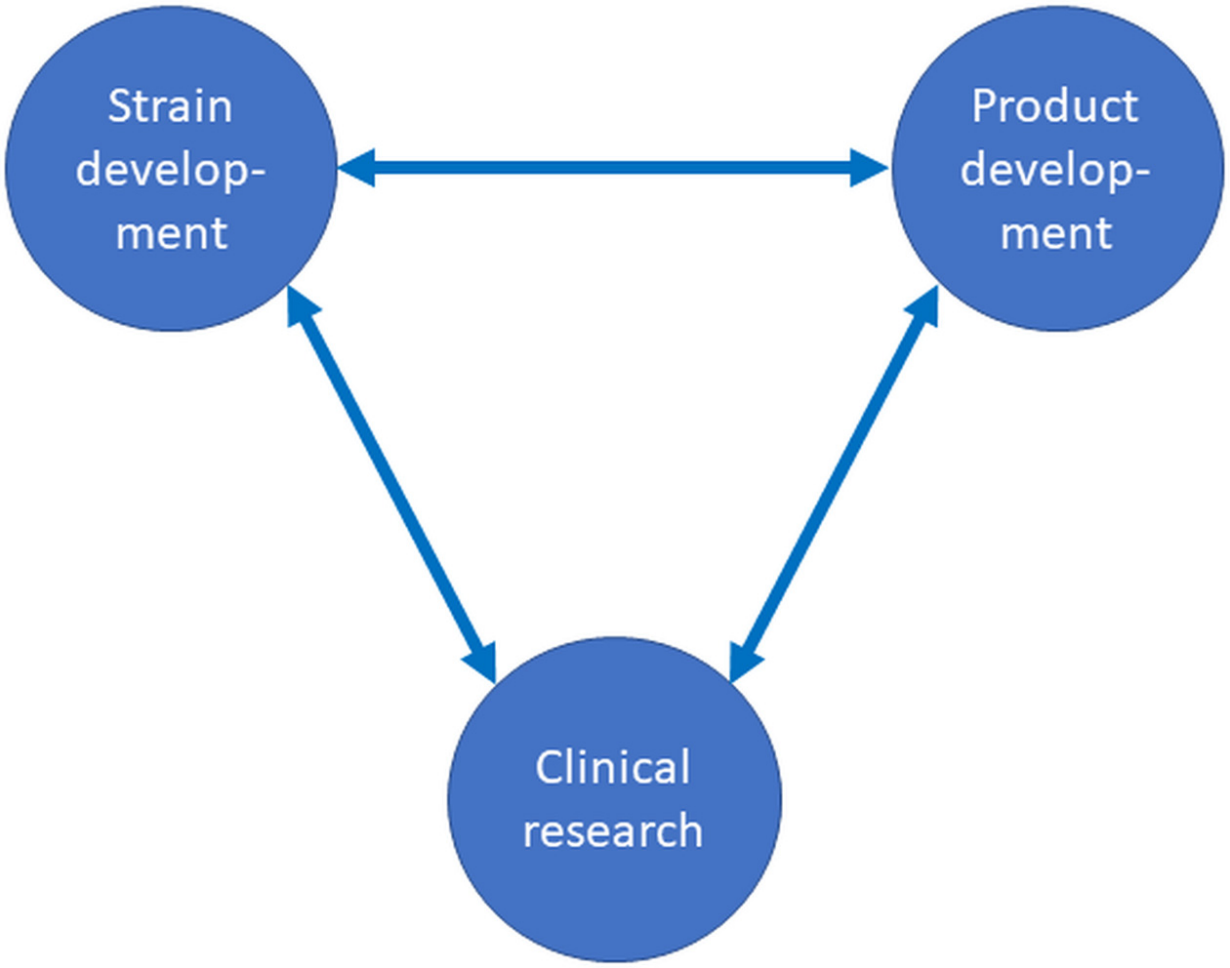
Schematic representation of the main areas involved in the development of probiotic products.

Probiotics are live microbes that, when administered in adequate amounts, confer a health benefit to the host (Hill *et al*. 2014). As was recently discussed, the definition centres around four criteria; (i) characterisation; (ii) safety for the intended use; (iii) clinical documentation and (iv) an efficacious dose throughout shelf life (Binda *et al*. 2020). Here, we want to focus on the applied side of probiotics; the development of the strain(s), the documentation of their health benefits and development of the commercial products (Fig. 1). These three areas are required to bring a successful probiotic product to the market and provide benefits to the consumer. We will focus on ‘conventional’ probiotics that are mainly sold as dietary supplements, ingredients of functional foods and beverages. Although probiotics are increasingly marketed in personal care products; such products usually contain lysates and/or metabolites from probiotics; so-called postbiotics and parabiotics (Martin and Langella 2019) and fall outside the scope of the present review. Live biotherapeutic products (LBPs) are a specific subclass of probiotics. As the name indicates these products aim a pharmaceutical status which has very different safety and efficacy requirements compared to conventional probiotics and will therefore not be included in the current review either. For more information on LBPs, the reader is recommended to consult other recent publications such as (Andrade *et al*. 2020; Rouanet *et al*. 2020). In 2016 the FDA issued a guidance document for LBPs, stipulating the manufacturing requirements necessary for probiotics intended to be studied as drugs in clinical trials (FDA 2016).

## STRAIN DEVELOPMENT

Technological demands placed on probiotics are substantial and often underestimated. Microbes of interest must be culturable under industrial conditions and they need to maintain viability, stability and functionality during the entire product shelf life.

**Figure 2.**
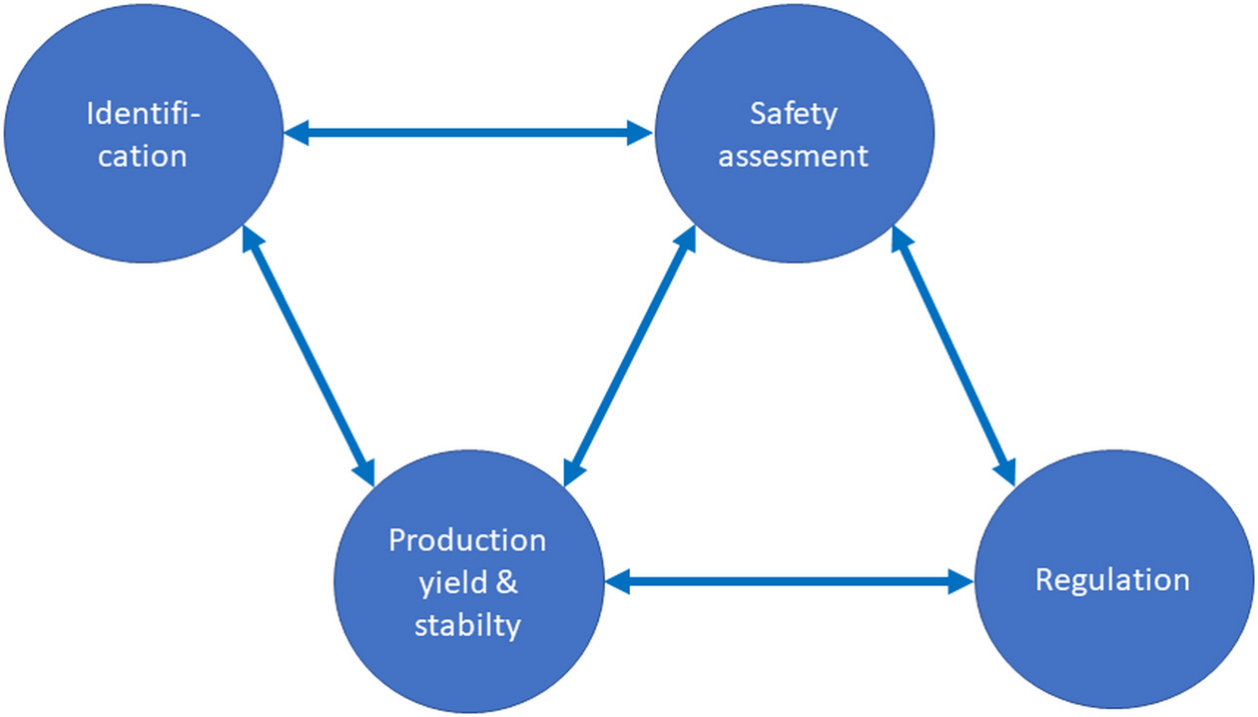
Schematic representation of the main factors influencing in the development of probiotic strains.

Obviously, the organisms must be safe for their intended use and fulfil all other requirements set by the local or regional regulatory bodies (Fig. 2). Most current probiotics belong to the genera *Bifidobacterium* and *Lactobacillus sensu lato*; although species from other genera such as *Bacillus*, *Saccharomyces* and *Escherichia* have also been documented as probiotic.

One of the first steps in the development of probiotic strains is identification. It is recommended that identification is performed by appropriate molecular techniques. Whole genome sequencing is the gold standard of identification and is recommended also by the European Food Safety Authority (EFSA) for bacteria and is nowadays very affordable. For yeasts, characterisation should be done by phylogenomic analysis; e.g. using a concatenation of several conserved genes (e.g. AFToL genes) to produce a phylogeny against available related genomes, 18S rDNA/ITS region or by alignment to a complete genome from the same species (EFSA 2020a). In communications and on commercial products, the proper nomenclature should be used following the International Code of Nomenclature. The strain name should consist of the genus and species name (and subspecies name when relevant) followed by a strain designation and/or culture collection number (Binda *et al*. 2020). It is therefore strongly recommended that the strain has been deposited in an internationally recognised culture collection. Currently there are 792 culture collections from 78 countries and regions registered (http://www.wfcc.info/ccinfo/, 30th of June 2020). Changes in nomenclature, such as the recent change in the family of *Lactobacillaceae* (Zheng *et al*. 2020), however, may require a transition period as regulators may need to update e.g. so-called positive lists.

Furthermore, proper identification is a first indicator for the safety of the organism. In the European Union a list of organisms presumed to be safe for human consumption is maintained and regularly updated by EFSA; the so-called Qualified Presumption of Safety (QPS) list (EFSA 2007, 2020b). However, if a strain does not belong to a QPS-listed species, it does not mean it is unsafe. It would be considered a Novel Food according to EU regulation (EU 2015). Other jurisdictions have other procedures to assess safety of probiotics, such as the Generally Recognized As Safe (GRAS) regulation in the United States. For ‘conventional’ probiotics belonging to the genera *Bifidobacterium* and *Lactobacillus sensu lato*, absence of undesirable transferable genetic properties, mainly antibiotic resistances, needs to be confirmed, also for strains from QPS-listed species. Standardised phenotypic tests with defined break points for antibiotic resistances exist and are maintained by EFSA (2018). If resistance above the break point is observed, further analyses are required. Here, the whole genome sequence is beneficial as a search can be done for putative resistance genes and if there are mobile elements in the vicinity that would potentially make such genes transferable. In addition, the whole genome sequence may help in documenting the presence or absence of other putative risk genes (Binda *et al*. 2020). For yeasts, the safety of potential probiotic strains or novel strains without safety documentation needs to be assessed. Both *in vitro* and *in vivo* animal models have been used for assessing safety. However, there are currently no established criteria for the safety testing of (potential) probiotic strains, and there is no regulatory model for approval of novel strains. The Panel on Additives and Products or Substances used in Animal Feed (FEEDAP) relies on the use of *in vitro* cell-based methods to detect evidence of potential cytotoxic effects (EFSA 2018).

The next big step is the production of the strain. For this, in an early stage it needs to be determined if a strain can be cultured on industrial scale, if it generates sufficient yield and it can be expected to have adequate stability. Some of these parameters can be determined in laboratory scale, such as growth requirements and an indication of yield and stability. However, due to the volume with which the industrial process operates, several factors cannot be simulated. The industrial process cannot be as rapidly stopped as in laboratory scale since cooling the whole production batch takes time, while the actual cooling through heat exchangers is actually very fast. Separation of the microorganisms from the medium takes time. Thus, there are hold-times that are not present and may be challenging to simulate at a small scale. The production therefore exposes the strain to various stresses which may affect survival and functionality (Fiocco *et al*. 2020). Although growth conditions and media can be tested at laboratory scale, great care must be taken with the choice of medium ingredients, cryoprotectants and lyoprotectants. Firstly, all the components must be food grade. Secondly, various national and regional regulators may have their own specific requirements. Further, customers may have requirements on the absence of certain allergens from the product. This may further restrict the choice of ingredients if there is a requirement for e.g. a dairy and soy free product.

The inoculum for the culture needs to come from a large stock frozen seed vials (Fenster *et al*. 2019). For culturing, it is important to reduce genetic drift; the number of generations during production therefore needs to remain as low as possible (Sanders *et al*. 2014). After culturing, the microbes need to be separated from the spent medium. Usually this separation is done by continuous centrifugation. The concentrate is then mixed with cryoprotectants and lyoprotectants. The former protects the cell against the damaging influence of freezing by controlling the formation of ice crystals. The latter protects the cell during the drying process (Fenster *et al*. 2019). Various processing aids have been studied and suggested to improve survival during processing and stability during storage as well as survival, and thereby potentially efficacy, during gastrointestinal passage (Upadastra *et al*. 2011). When probiotics are intended to be added to e.g. dairy products, juices, etc. it may be sufficient to only freeze the concentrated material. However, if the probiotics are to be included in dietary supplements such as capsules or sachets or are included in dry delivery formats such as infant formula, the concentrated material needs to be dried. The most economic method of drying is spray drying. However, due to the relatively high temperature, this is not the preferred method for most probiotics. Instead, freeze drying is the most commonly applied method of drying probiotics (Broeckx *et al*. 2016). After drying, the product can be milled to a desired particle size and can be standardised to a given count with suitable excipients, e.g. microcrystalline cellulose or maltodextrin (Forssten, Sindelar and Ouwehand 2011). Thereafter the product is ready to be incorporated into clinical study materials and/or commercial products.

## CLINICAL RESEARCH

For a microbial strain or a combination there of to be considered probiotic, it must have shown its effect in a human intervention trial. Randomised, double-blind, placebo-controlled and registered human clinical trials are at the core of probiotic innovation and ensure reliable and transparent research (Dronkers, Ouwehand and Rijkers 2020).

**Figure 3.**
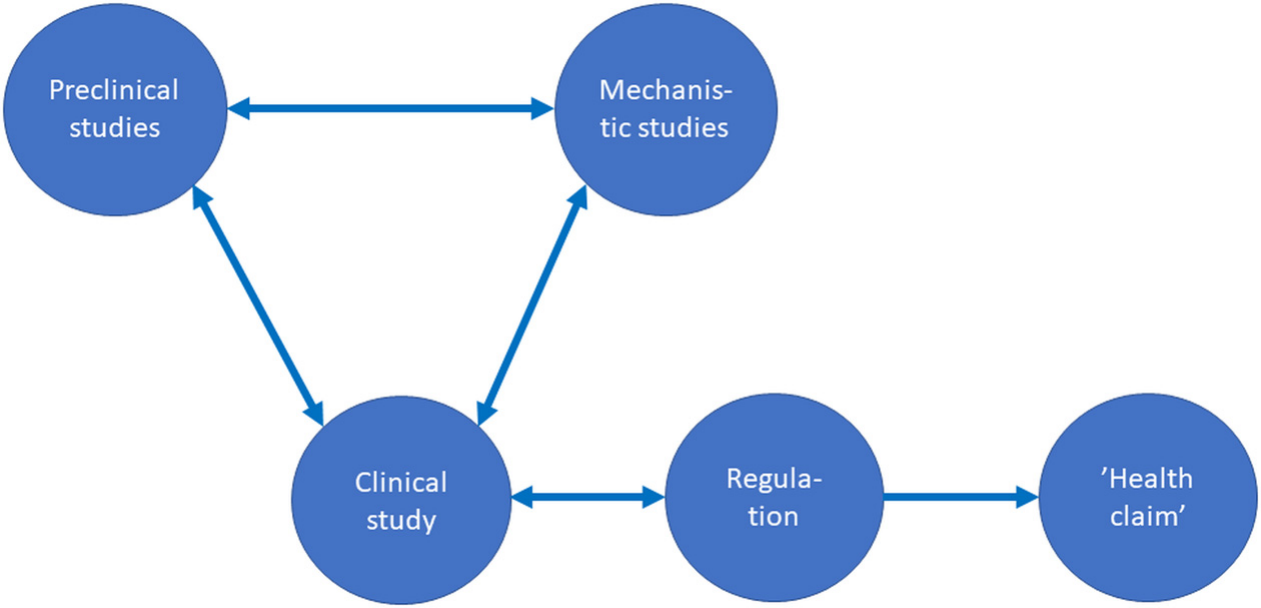
Schematic representation of the main factors influencing clinical studies with probiotics and subsequent ‘claiming’ of the outcome.

In the initial stages, the probiotic potential of a strain or a strain combination can be studied by *in vitro* methods (Fig. 3). A total of two distinctive traits of a potential probiotic strain are the tolerance to low pH and bile acids making it more likely for the strain to remain viable and survive the passage through the harsh environment of the gastrointestinal tract (Vizoso Pinto *et al*. 2006). Most of the studies have used methods where probiotics have been exposed to a constant low pH (1.5–3), although it would be better to mimic the gradual increase of acidity occurring in the gastrointestinal tract (Burns *et al*. 2014). In order to further assess the specific activity of a strain, different *in vitro* methods can be used. By using an assay for stimulation of immune cells it can be verified if strains can modulate immune activities (Maldonado Galdeano *et al*. 2019). It may also be relevant to identify whether the strains can produce antimicrobial compounds including bacteriocins as well as adherence properties of the strain. Adherence to Caco-2 cells can be used as a competition assay for pathogen adhesion. Although adherence of probiotics to the intestinal epithelium may contribute to their persistence on the mucosal surface, the gastrointestinal colonisation of orally administered probiotics seems to be temporary (Taverniti *et al*. 2019). Even though Caco-2 cells are widely applied, they are not perfect as an *in vitro* model to study adhesion and the mechanisms of action of probiotics. Being a cancer cell line, they have different properties e.g. glycosylation as compared to normal intestinal cells. In addition, they represent a combination of both large- and small-intestinal epithelial cell phenotypes (Brooks *et al*. 2008). Thus, the attachment results of probiotics to monolayers of epithelial cells may not always give the complete picture for adhesion occurring *in vivo*. Human intestinal tissue pieces representing the local microbiota can be used for adhesion assessment, and mimic conditions in different parts of the intestine (Ouwehand *et al*. 2002). If selecting e.g. disease-specific probiotics this type of model could be used, since most methods do not include the presence of the normal microbiota (Vinderola *et al*. 2017). In addition, computer controlled models can be used for studying survival through the gastrointestinal tract, and in these a ’normal’ microbiota can also be present (Van de Wiele *et al*. 2015; Forssten and Ouwehand 2020).

Different *in vivo* models are used to study the capabilities of probiotics. For example, different mouse models have been widely applied to study the mechanisms underlying the pathogenesis. Recently *Caenorhabditis elegans* (a nematode model) has become an increasingly valuable *in vivo* model for studying interactions between the host and the probiotic strain (Guantario *et al*. 2018). *C. elegans* is a nematode with a transparent body, short lifecycle, small size and absence of ethical issues. In addition, it is suitable for screening studies and inexpensive to maintain (Clark and Hodgkin 2014). *C. elegans* is a powerful tool to test the effects of ingested bacteria on host physiology and it can also be useful in providing mechanistic insights on the beneficial effects of probiotics (Roselli *et al*. 2019; Goya *et al*. 2020).

Despite the above described models, in the end health efficacy can only be supported by clinical studies (Fig. 3). Therein such studies, the study design, objectives, number of study participants, duration of study, monitoring and passive surveillance of the study as well as the data collection and the analyses performed all impact on the study results. Clinical trials can be either run as a single centre or multicentre studies. However, the number of sites should not impact on the study results. For some indications it might be necessary to perform a multicentre study e.g. due to a rare medical endpoint, or to speed-up the time course for a study.

Since the diversity of the microbiota varies between individuals and is influenced by diet, lifestyle, health status, etc. it is essential to select subjects that are most likely to benefit from the study. In other words, it is important to define a healthy or dysbiotic microbiota, to have health endpoints that follow regulatory guidance and to be able to have measurable restoration of the microbiota. Similar considerations should be made for other primary endpoints as well and at the same time indicate the weakness of secondary endpoints; the population may be suboptimal for those. In addition, health efficacy studies for probiotics have numerous challenges due to multiple influences on the microbiota and other endpoints. Such influences e.g. subject variation, changes in lifestyle and diet, use of medications, etc. can lead to increased variability and associated challenges in reproducing the data. However, by developing a clear clinical protocol describing how the clinical trial will be conducted considering the objective(s), design, methodology, statistical considerations and organisation of the trial, both ensures the safety of the trial subjects as well as the integrity of the data collected. In addition to the protocol itself it is also advised to have a data management plan (DMP) to verify high quality of the clinical trial data. The DMP confirms the process of collection, cleaning and management of subject data in compliance with regulatory standards (Ohmann *et al*. 2011). Thus, the DMP provides high-quality data by limiting the number of errors and missing data, i.e. to gather maximum of complete and reliable data to be included for analysis.

Another important aspect of a clinical trial is the active documentation of safety data. The documentation of adverse events provides a reliable safety profile of the investigated product since all adverse events are recorded and not only those events that an individual investigator considers to be related to the study product based upon her/his own individual judgment (Glikich, Dreyer and Leavy 2014). Moreover, the safety data linked to administration of a study product to heathy volunteers can also be important to identify adverse events related to the study product before proceeding with studies in more vulnerable populations.

The dose of the probiotic is an important point of consideration. Consumption of a higher dose not necessarily have a greater health benefit than a lower dose. One advantage of probiotics as compared to intake of metabolite-based products is the relatively longer half-life, (Koh *et al*. 2016). In general, dose-ranging studies with probiotics are rare. In practice, most clinical studies with probiotics use doses between 10^8^ and 10^11^ colony forming units (CFU). This seems to be an efficacious, safe and economical dose range (Ouwehand 2017).

In addition to results from preclinical and clinical study results, advances in machine learning algorithms can be applied to large data sets. Hence, it is possible to further study the complex interactions between the microbiome and the host and thereby the efficacy of a probiotic or clinically meaningful patterns in the data can be identified (Shah *et al*. 2019). If metabolic, demographic and other data sets are combined with the phenotypic traits and the microbiome from various studies, it might be possible to identify potential probiotic strain (combinations) and thereby reduce time and cost of probiotic screening (van den Bogert *et al*. 2019).

Meta-analyses are also used in order to evaluate the value of probiotics in e.g. randomised controlled trials. The downside with both meta-analyses and reviews is that in general data from studies using different probiotic species might be pooled and thus the outcome might be different to the conclusions published for randomised controlled trials. However, this is not necessarily ambiguity since it can indicate that a specific probiotic species did not work under the specified conditions (McFarland, Evans and Goldstein 2018). Thus, it is important to remember that generalisation of the efficacy of probiotics should be avoided. A total of two bacterial strains with small differences in the genome sequence, although otherwise genetically nearly identical, may have different phenotypes and thereby leading to different outcomes *in vivo (*Zeevi *et al*. 2019*)*. Further, with an increasing number of studies, meta-analyses are starting to be feasible on specific highly-investigated strains. Health efficacy should preferably be confirmed in independent trials studying a specific probiotic strain/strain combination in a specified population.

Clinical studies are thus needed to document that a strain is probiotic. Further, it also serves to market the product that contains the probiotic. However, what can be ‘claimed’ is strictly regulated. In the case of foods and dietary supplements, as discussed here, no statements can be made suggesting, to cure, prevent, mitigate or diagnose disease as such claims are reserved for drugs. What can be claimed is regulated on a national or regional level. In the US, so-called content and structure-function claims are allowed, while in the EU only claims allowed by the European Commission can be made. In the EU, dossiers for health claims are evaluated by EFSA. To date (1st October 2020) no positive opinion has been given by EFSA for any of the submitted probiotic dossiers. In its evaluation, EFSA focusses on four main points:

1. Is the food/constituent (i.e. the strain) defined and characterised?
2. Is the claimed effect defined and is it a beneficial physiological effect for the target population, and can be measured *in vivo* in humans?
3. Has a cause and effect relationship been established between the consumption of the food/constituent (i.e. the strain) and the claimed effect?
4. Is a plausible mechanism of action proposed?

While early dossiers, due to unfamiliarity with the application process, failed at all points. Recent dossiers have not succeeded in convincing EFSA on point 3, the cause and effect relationship (EFSA panel on dietetic products 2016). Alternative approaches have been pursued, using e.g. the high vitamin B12 or vitamin K2 content of selected propionibacteria and lactococci. These vitamins have an approved EU health claim. However, strains as sources of such vitamins have not be evaluated by EFSA and as such do therefore not have a positive EFSA opinion.

Finally, in any case, regardless of the jurisdiction, a manufacturer is never allowed to mislead the consumer.

## PRODUCT DEVELOPMENT

**Figure 4.**
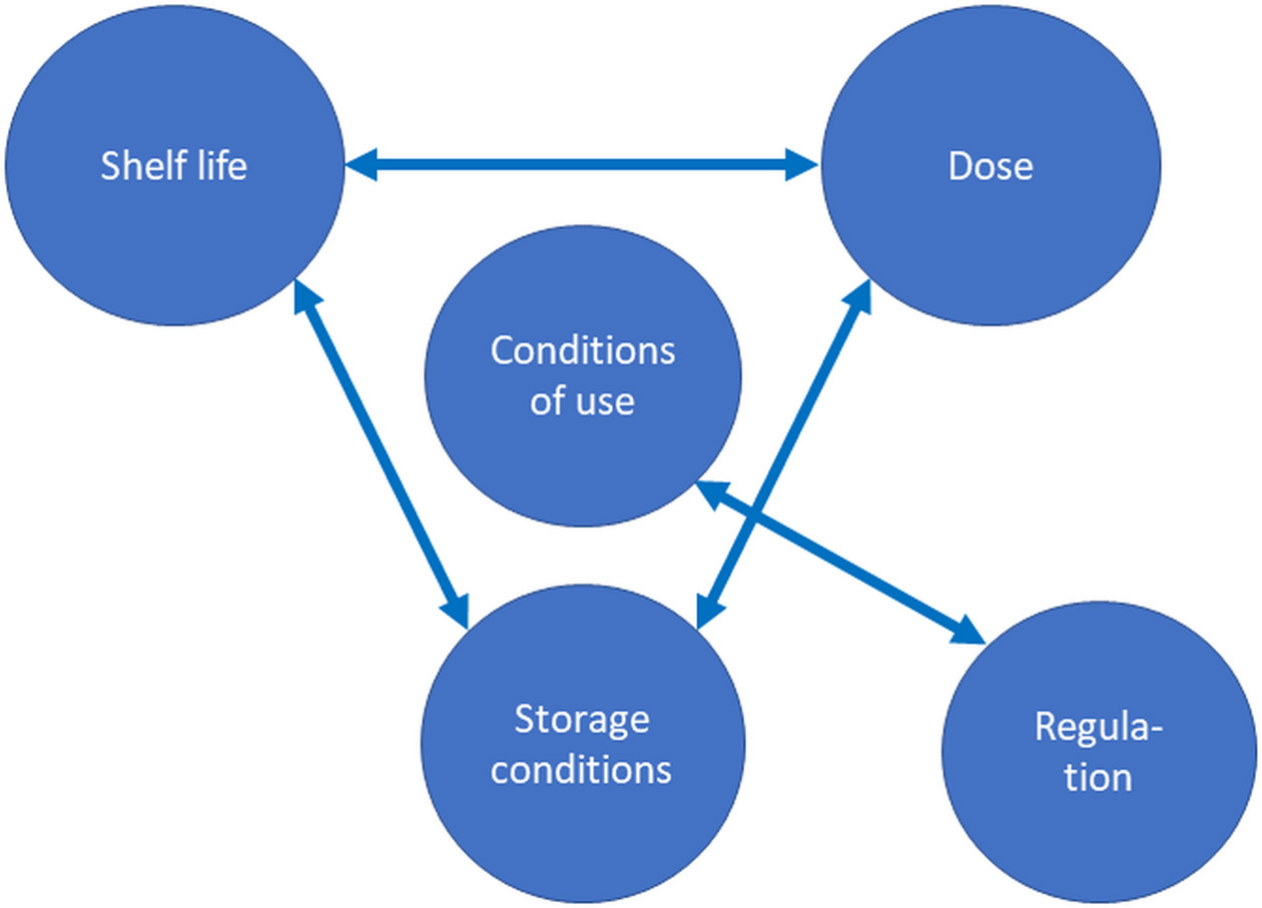
Schematic representation of the main factors influencing in the development of probiotic products.

The development of probiotic strains and the documentation of their health benefits are of no use if they cannot be brought to the consumer. In general, probiotics are mostly marketed as either functional foods or dietary supplements. In either case, the challenge is to provide the efficacious dose at the end of shelf-life. This dose of live microbes would be the same dose as the one studied and shown to provide a health benefit in a human clinical trial, as described above. Because probiotic viability will decrease over time, an overage of the strain/s may be required. The viability and thereby the shelf-life is influenced by the storage conditions and there are regulatory requirements to be complied with (Fig. 4).

There are two main factors influencing stability of microorganisms in general and probiotics in particular; water activity (a_w_) and temperature. In products with a high moisture content, environmental factors such as pH and antimicrobials may influence survival (Forssten, Sindelar and Ouwehand 2011).

Functional foods can be divided into high and low-moisture products. High-moisture products are e.g. probiotic fermented dairy products or probiotic juices. These are commonly stored refrigerated and have a shelf-life of 4–8 weeks. Low-moisture products are e.g. granola bars and infant formula. Similar to dietary supplements, which tend to be low-moisture products, such as capsules and sachets, the storage typically is at ambient temperatures for up to 24 months (Forssten, Sindelar and Ouwehand 2011; Fenster *et al*. 2019).

In high-moisture products it is important that probiotics have no negative impact on the product formulation, this is specially the case with foods and beverages. Uncontrolled metabolic activity, such as post-acidification, may cause unwanted changes in end-product structure or flavour. Further, in high-moisture products a low pH requires consideration. Even though probiotics are commonly selected to tolerate low pH, as outlined above, an acidic product may negatively affect the survival of the probiotics. Some products, such as fruit juices, may contain naturally occurring antimicrobial components that influence the survival. Inclusion of probiotics in fruit juices may thus require particularly robust strains or probiotic strains require extra protection. The stresses to which probiotic strains may be exposed to during food production and storage may not only affect their survival, but also their efficacy. Methods to improve resistance to environmental stresses include, microencapsulation, protective carrier media, prebiotics, stress adaptation and cross-protection, selection of resistant strains and genetic engineering or adaptive evolution (Fiocco *et al*. 2020).

In low-moisture products it is important to maintain the low moisture; an a_w_ of 0.20 or even 0.15 is recommended for long-term storage (Fenster *et al*. 2019). In functional foods, this can be accomplished by including the probiotic in a hydrophobic carrier such as chocolate (Possemiers *et al*. 2010). It is also important to control the a_w_ of the food and be aware of the possible hygroscopicity of food ingredients. In general, the use of appropriate packaging with a good moisture barrier function is beneficial. For dietary supplements, dry excipients should be used in the formulation. Here too, hygroscopicity may be a challenge, this is particularly so for the formulation of synbiotics. The prebiotic component often has a high hygroscopicity and may thus negatively affect the survival of the probiotic component in the formulation. Packaging can provide a good moisture barrier: glass bottles provide the best barrier, but an alternative solution is the use of HDPE bottles with a desiccant in the bottle wall (CSP® brand vials). These bottle types have proven to be highly effective at maintaining the low a_w_ of the probiotic contents throughout shelf life, even at high-humidity storage conditions (Fenster *et al*. 2019).

Although not required in the definition, there is a common expectation that probiotics are able to survive and (at least transiently) colonise the gastrointestinal tract. To what extent the food matrix influences this, is still a matter of debate. However, it is important to realise that probiotics are consumed as part of a diet and not as a single food matrix or dietary supplement. The effect of the diet on probiotic survival may very well dwarf the effect the probiotic delivery format potentially has. Early studies on faecal recover did not observe difference in faecal probiotic levels, regardless whether *Lacticaseibacillus rhamnosus* GG or *L. rhamnosus* Lc705 were consumed as capsule, yogurt or cheese. However, *Propionibacterium freudenreichii* ssp. *shermanii* JS survived best when administered in capsule or yoghurt, while *Bifidobacterium animalis* ssp. *lactis* Bb-12 survived best when administered in yogurt. To what extent these differences translate in a potential difference in efficacy remains to be determined (Saxelin *et al*. 2010). In the case of dietary supplements, strains with a poor acid and bile resistance can be protected using enteric coated capsules (Tan *et al*. 2019).

Quality control and quality assurance are essential in the manufacturing of all products, and probiotics are no exception. Probiotic products need to comply with national/regional regulations for their intended use. It is a common, but unfortunate, misunderstanding that probiotics are not regulated or regulated at a lower standard then drugs. This is not the case, they are regulated at a different standard, usually a food standard. In practice, this may even be a stricter regulation as for food there is very little tolerance for potential risks, while for drugs a considerable risk may be accepted as specific substantial benefits are expected.

Thus, the production of a good quality efficacious probiotic requires substantial consideration. As with the health benefits, also stability is largely strain dependent, although some species tend to be more robust than others.

## CONCLUSIONS

The manufacturing of a successful probiotic product involves several critical steps. From the choice and production of the strain via the conduct of preclinical and especially clinical trials to the production of the consumer product. All steps need to be considered and successfully taken. Failure to do so may lead to serious challenges later in the production process. When these steps have been taken, they form the ground for successful innovations and provide end-user acceptance and trust. Open communication and consumer education are the final essential steps in successful probiotic product development for future consumer needs and expectations. All of this must happen within the national or regional regulatory framework where the probiotics are manufactured, studied and commercialised.
